# Comprehensive detection of recurring genomic abnormalities: a targeted sequencing approach for multiple myeloma

**DOI:** 10.1038/s41408-019-0264-y

**Published:** 2019-12-11

**Authors:** Venkata Yellapantula, Malin Hultcrantz, Even H. Rustad, Ester Wasserman, Dory Londono, Robert Cimera, Amanda Ciardiello, Heather Landau, Theresia Akhlaghi, Sham Mailankody, Minal Patel, Juan Santiago Medina-Martinez, Juan Esteban Arango Ossa, Max Fine Levine, Niccolo Bolli, Francesco Maura, Ahmed Dogan, Elli Papaemmanuil, Yanming Zhang, Ola Landgren

**Affiliations:** 1 Myeloma Service, Department of Medicine, Memorial Sloan Kettering Cancer Center, New York, NY USA; 2 Department of Medicine Solna, Karolinska Institute, Stockholm, Sweden; 3 Cytogenetics Laboratory, Department of Pathology, Memorial Sloan Kettering Cancer Center, New York, NY USA; 4 Bone Marrow Transplant Service, Department of Medicine, Memorial Sloan Kettering Cancer Center, New York, NY USA; 5 Center for Hematological Malignancies, Department of Medicine, Memorial Sloan Kettering Cancer Center, New York, NY USA; 6 University of Milan, Department of Oncology and Onco-Hematology, Milan, Italy; 7 Fondazione IRCCS Istituto Nazionale dei Tumori, Department of Medical Oncology and Hematology, Milan, Italy; 8 Hematopathology Laboratory, Department of Pathology, Memorial Sloan Kettering Cancer Center, New York, NY USA; 9 Epidemiology & Biostatistics, Department of Medicine, Memorial Sloan Kettering Cancer Center, New York, NY USA

**Keywords:** Genetics research, Cancer genomics

## Abstract

Recent genomic research efforts in multiple myeloma have revealed clinically relevant molecular subgroups beyond conventional cytogenetic classifications. Implementing these advances in clinical trial design and in routine patient care requires a new generation of molecular diagnostic tools. Here, we present a custom capture next-generation sequencing (NGS) panel designed to identify rearrangements involving the *IGH* locus, arm level, and focal copy number aberrations, as well as frequently mutated genes in multiple myeloma in a single assay. We sequenced 154 patients with plasma cell disorders and performed a head-to-head comparison with the results from conventional clinical assays, i.e., fluorescent in situ hybridization (FISH) and single-nucleotide polymorphism (SNP) microarray. Our custom capture NGS panel had high sensitivity (>99%) and specificity (>99%) for detection of *IGH* translocations and relevant chromosomal gains and losses in multiple myeloma. In addition, the assay was able to capture novel genomic markers associated with poor outcome such as bi-allelic events involving *TP53*. In summary, we show that a multiple myeloma designed custom capture NGS panel can detect *IGH* translocations and CNAs with very high concordance in relation to FISH and SNP microarrays and importantly captures the most relevant and recurrent somatic mutations in multiple myeloma rendering this approach highly suitable for clinical application in the modern era.

## Introduction

Multiple myeloma is a heterogeneous disease in terms of genomic alterations, clinical presentation, and survival outcomes. The genetic landscape in multiple myeloma is complex and historically includes two main categories of abnormalities: hyperdiploidy, defined as gains of odd numbered chromosomes, and immunoglobulin heavy chain (*IGH*) translocations, including t(4;14), t(6;14), t(11;14), t(14;16), and t(14;20)^1–3^. In addition, recurrent chromosomal gains and losses have been reported, e.g. gain 1q, del(13q), and del(17p)^2,3^. Some of these aberrations define subgroups of patients associated with poor prognosis in the majority of published studies, e.g. t(4;14), t(14;16), and del(17p)^1,4^.

The advent of next-generation sequencing (NGS) has progressively expanded our knowledge of multiple myeloma biology identifying new and recurrent driver events such as single-nucleotide variants (SNVs) and focal deletions^5–9^. Emerging data suggest that the current high-risk definition can be further improved by integration of recurrent mutations and distinct cytogenetic profiles with the International Staging System^10–14^. Specifically, bi-allelic events including *TP53* and more than three copies of 1q have been recently associated with poor outcomes^5,8,15^.

Currently, in the standard of care setting, conventional chromosome analysis, multiple myeloma targeted fluorescence in situ hybridization (FISH) panels, and single-nucleotide polymorphism (SNP) microarrays are used to detect chromosome translocations and gains and losses in multiple myeloma^2,3^. Conventional chromosome analysis is labor intensive, has low genomic resolution, and is often inadequate due to low mitotic activity and low percentage of plasma cells in bone marrows^16^. Although FISH is accurate to define distinct recurrent aberrations, it is limited to the targets of the selected probes not allowing a comprehensive cytogenetic characterization. SNP microarrays have been used to summarize copy number changes in multiple myeloma. This approach still relies on FISH for the detection of *IGH* translocations and usually requires about 15–20% or more aberrant plasma cell infiltration of the bone marrow which in turn limits the availability to do parallel comprehensive genomic analysis including V(D)J profiling for minimal residual disease characterization/tracking^17,18^. Furthermore, neither FISH nor SNP microarray approaches are able to capture somatic point mutations.

We were motivated to develop a new targeted NGS assay designed to capture frequently defined multiple myeloma subtypes. Specifically, the assay captures recurrent *IGH* translocations, arm level, and focal copy number alterations (CNAs), as well as frequently mutated genes in multiple myeloma ur aim was to develop a novel strategy that could replace current standard of care assays and to capture mutational status in multiple myeloma in a single assay. In a head-to-head comparison with standard of care FISH and SNP microarrays, our assay revealed an extremely high concordance (sensitivity > 99% and specificity > 99%) for both *IGH* translocations and CNAs. Additionally, we captured mutations and bi-allelic events relevant to clinical outcomes in multiple myeloma. Our study provides strong evidence that multiple myeloma designed custom capture NGS panels are of directly clinical relevance and can be used as a novel strategy to replace the current standard of care techniques.

## Methods

### Custom capture next-generation sequencing panel

In this head-to-head comparison of standard of care multiple myeloma genomic sub-typing assays (i.e. FISH and SNP microarrays) and NGS-based genomic sub-typing, we used an in-house developed multiplex custom capture NGS assay (named “myTYPE”) which has been designed to detect the most common and relevant genomic aberrations in multiple myeloma^5–9,19,20^. To capture *IGH* (14q32) rearrangements, we included the canonical *IGH* locus. CNAs are assessed through genome-wide representation of SNPs, one in every 3 Mb, to enable detection of CNAs. To capture focal events, SNPs were tiled at a higher density in loci and genes which are commonly deleted or amplified in multiple myeloma^3^. Additionally, we included 120 genes that were selected on the basis of (a) genes frequently mutated in multiple myeloma from earlier reports^5–7,21–26^, (b) genes that are involved in important signaling pathways in multiple myeloma, e.g. the MAPK and NFKB pathways (Supplementary Table)^6^, (c) treatment targets and candidate genes for drug resistance in multiple myeloma (e.g., *CRBN*, *IKZF1*, and *IKZF3)*^27^, and (d) candidate genes and SNPs associated with an increased susceptibility of developing multiple myeloma^28^. The total target space was 2.06 Mb. The final bait design was created using Nimblegen SeqCap.

### Patient cohort

We identified 154 patients with plasma cell disorders who presented to Memorial Sloan Kettering Cancer Center (MSKCC) between 2015 and 2018 and had samples collected for conventional cytogenetic evaluation as well as samples collected under the MSKCC’s institutional tissue acquisition protocol. The bone marrow aspirate specimens underwent CD138-positive enrichment through magnetic bead sorting and were divided for FISH and SNP microarray testing. DNA was extracted using commercial Qiagen DNA extraction kits and used for SNP microarray and sequencing with the custom capture NGS panel. In addition, 16 unmatched bone normal marrow samples from healthy donors were used as assay controls to filter sequencing and chemistry specific artifacts and germline variation. All patients had consented to MSKCC’s institutional tissue acquisition protocol and the study was approved by the MSKCC Institutional Review Board.

### FISH and SNP microarray

FISH panels for multiple myeloma included probes for t(4;14), t(6;14), t(8;14), t(11;14), t(14;16), and t(14;20) from Abbott Molecular, Des Plaines, IL and Metasystems, Newton, MA. All FISH testing was performed in the MSKCCs clinical Cytogenetics Laboratory. Between 100 and 500 cells, if available, were analyzed. FISH probes for translocations t(4;14) and t(11;14) were tested in all patients while in six patients, there was not enough cell material to test for t(6;14), t(8;14), t(14;16), and t(14;20).

SNP microarrays with 2.67 million probes including 750 thousand common and rare SNP probes (Cytoscan, Affymetrix, Santa Clara, CA) were performed following manufacturers protocol. Data analysis was performed using Affymetrix ChAS 3 software and ASCAT^29^. Copy neutral loss of heterozygosity (CN-LOH) was reported if the size was at least 10 Mb at a terminal region or 20 Mb for an interstitial CN-LOH. For CNA comparison in this study, only commonly recurrent multiple myeloma aberrations: del(1p), gain 1q, del(6q), del(8p), del(13q), del(14q), del(16q), del(17p), and hyperdiploidy, were evaluated.

### Custom capture NGS panel data generation and processing

After DNA extraction, barcoded sequence libraries (New England Biolabs, Kapa Biosystems, Wilmington, MA, USA) were subjected to capture by hybridization (Nimblegen SeqCap, Madison, WI, USA). Between 100 and 200 ng of gDNA was used as input for library construction. DNA was subsequently sequenced on an Illumina HiSeq 2500 sequencer to generate paired-end 101-bp reads.

Translocations were analyzed using the algorithms BRASS and Delly^30,31^. CNVKit and FACETS were used to identify somatic CNAs^32,33^. Together with results from CNVKit, B-Allele frequencies using SNPs from the 1000 genomes project were used to identify regions of CN-LOH. The 16 unmatched control samples were combined into a pooled reference for CNA comparison. SNVs and indels were called combing results from CaVEMan, Strelka2, Mutect2, and Pindel^34–37^. Bi-allelic inactivation was assessed in patients with multiple myeloma and was defined as having a CNA, e.g. arm level or focal deletion, and somatic mutation affecting the same gene. Tumor-specific *IGH*-V(D)J rearrangement sequences were identified using the MiXCR algorithm^38,39^. Detailed information on variant calling is given in the Supplementary Material.

## Results

### Patient cohort

**Fig. 1:**
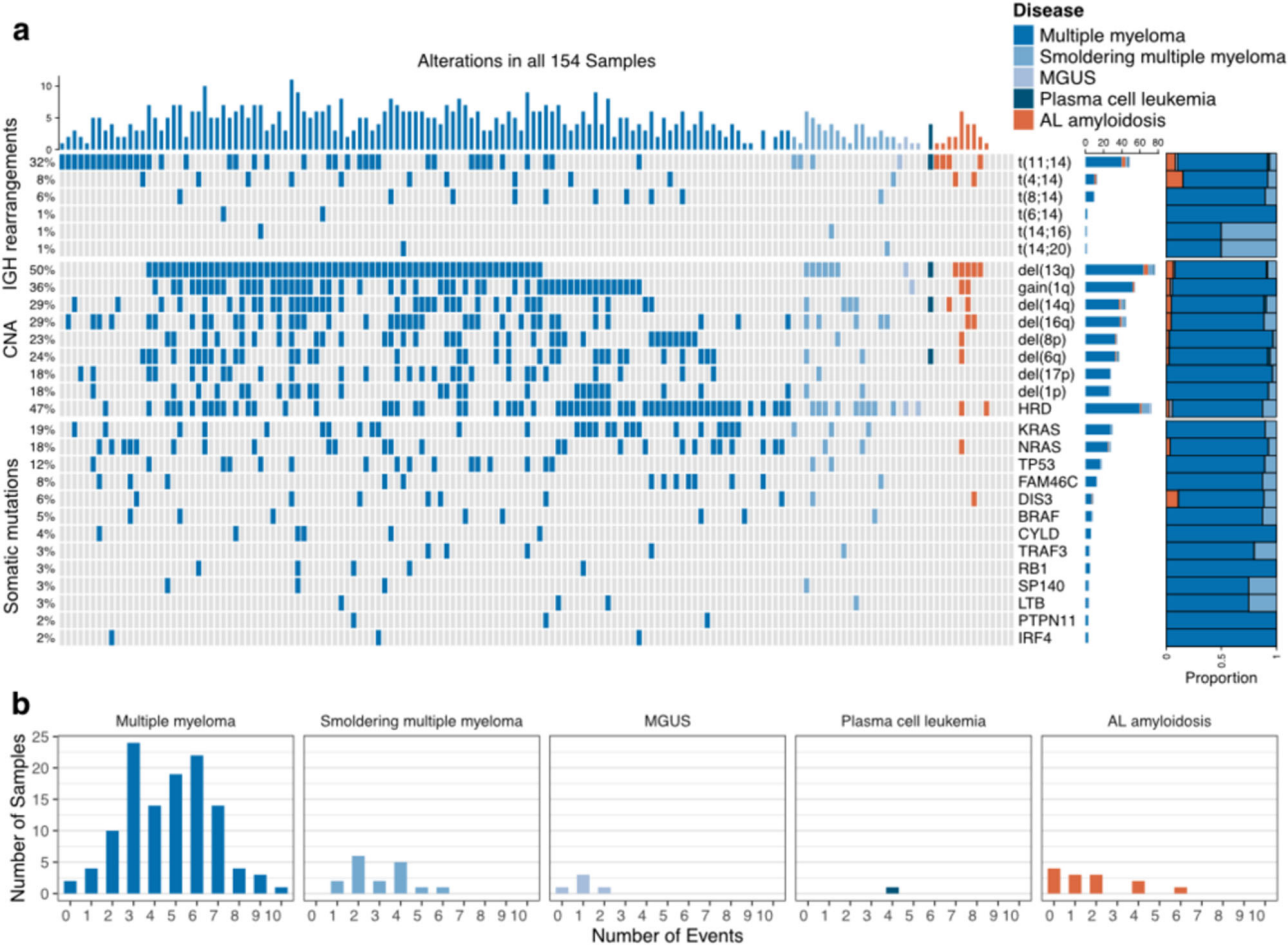
**a** *IGH* translocations, copy number alterations, and somatic mutations captured with myTYPE targeted sequencing. **b** Number of events in relation to disease status.

Bone marrow aspirates from 154 patients with plasma cell disorders; multiple myeloma (*N* = 118), smoldering multiple myeloma (*N* = 17), monoclonal gammopathy of undetermined significance) (*N* = 5), plasma cell leukemia (*N* = 1), and light-chain (AL) amyloidosis (*N* = 13) were included in the study (Table 1). With our custom capture NGS assay (myTYPE), *IGH* translocations were detected in 51%, multiple myeloma relevant CNAs in 83%, and somatic mutations in 85% percent of patients (Fig. 1).

**Table 1:** Patients’ characteristics.

Total, *N* (%)	154 (100%)
Median age, years (range)	63 (35–85)
Male sex, *N* (%)	76 (49%)
Diagnosis	
Multiple myeloma, *N* (%)	118 (76%)
Smoldering multiple myeloma, *N* (%)	17 (11%)
Monoclonal gammopathy of undetermined significance, *N* (%)	5 (3%)
Plasma cell leukemia, *N* (%)	1 (1%)
AL amyloidosis, *N* (%)	13 (8%)

### Assay coverage and sample purity

Sequencing using the custom capture NGS assay generated a median of 38 million paired-end reads per sample resulting in a median target coverage of 651x per sample (Supplementary Fig. S1). The coverage across the *IGH* locus, exonic regions, genome-wide copy number SNPs, and finger printing SNPs were homogeneous (Supplementary Fig. S2).

**Fig. 2:**
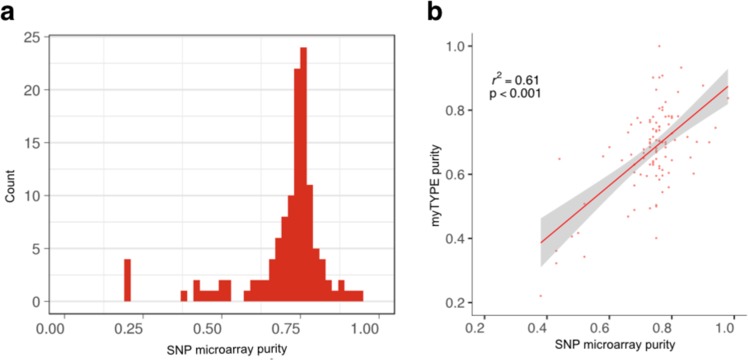
**a** Sample purity estimates SNP microarray and **b** correlation between sample purity estimated by SNP microarray and myTYPE sequencing.

The sample purity for SNP microarray was estimated using ASCAT and for the custom capture NGS assay using algorithms based on mutation calls. Median purity was 75% using ASCAT. There was a high concordance between the sample purity for the two methods (Pearson’s *r*^2^ = 0.61) (Fig. 2).

### Comparison myeloma targeted FISH versus the custom capture NGS assay

**Fig. 3:**
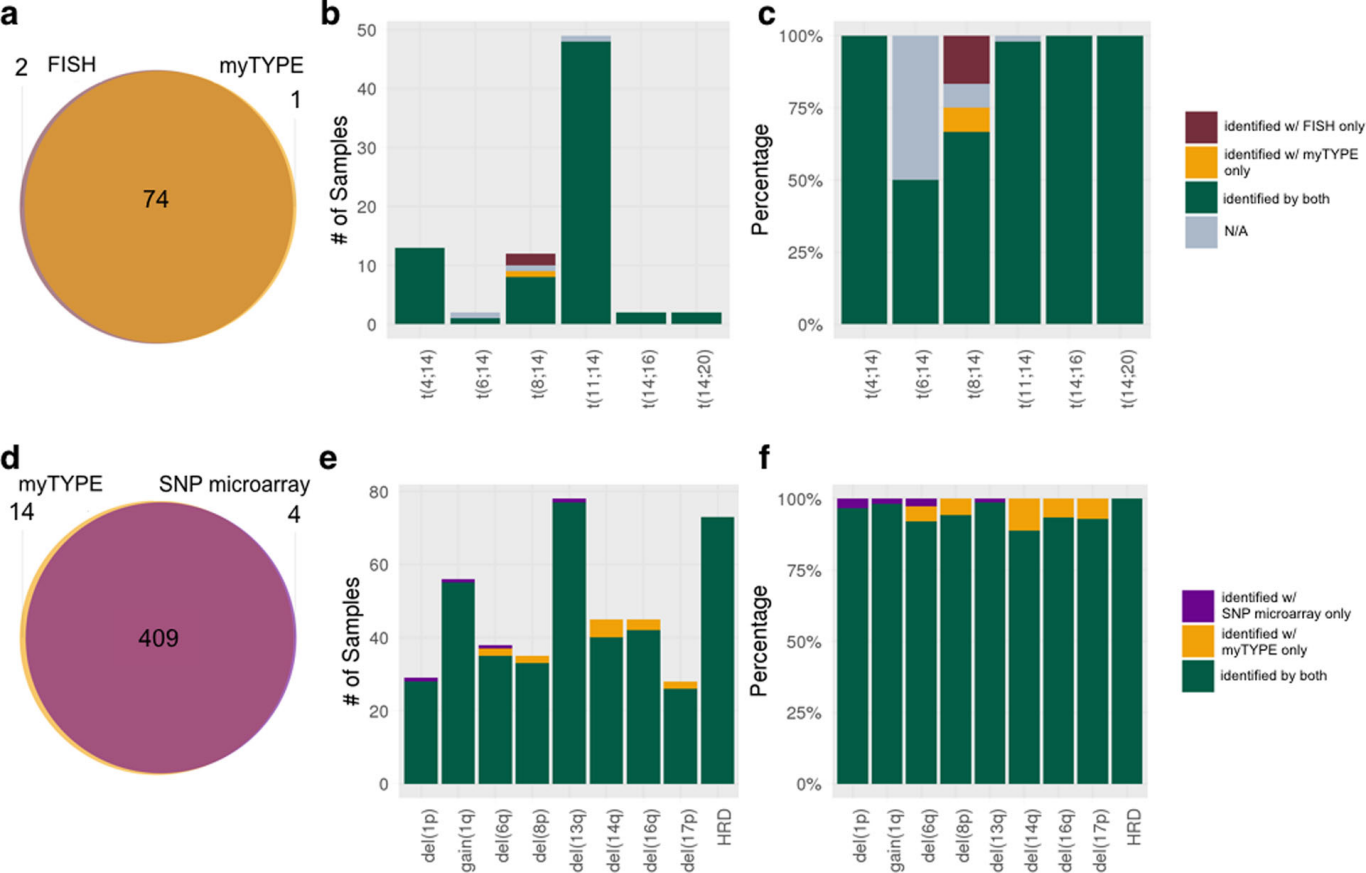
Concordance and discordance for the detection of genomic aberrations: multiple myeloma targeted FISH and SNP microarray versus myTYPE.

**Fig. 4:**
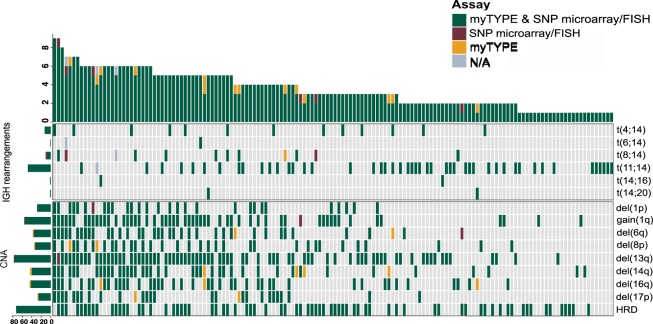
Comparison of multiple myeloma targeted FISH and SNP microarray versus myTYPE.

Across the 154 samples that were assessed with multiple myeloma targeted FISH and our custom capture NGS assay, 78 *IGH* translocations were detected by both approaches with a 100% concordance for t(4;14), t(6;14), t(11;14), t(14;16), and (14;20) (Table 2). One patient had both t(11;14) and t(8;14) translocation detected using the custom capture NGS assay, only the t(11;14) translocation was detected with FISH in this sample (Supplementary Fig. S3). In two patients, t(8;14) detected by FISH was not confirmed by our custom capture NGS assay. This may be indicative of variable *IGH* breakpoints in *MYC* translocations, which may be outside of the custom capture NGS assay’s primer coverage (Supplementary Fig. S4). The concordance was 96% (*N* = 75) with a sensitivity and specificity of 97% and 100%, respectively, for the custom capture NGS assay using FISH as the reference. The distribution and concordance of each of the translocations in the study cohort is given in Table 2 and Figs. 3 and 4.

**Table 2:** Common genomic aberrations and their detection rates using the custom capture next-generation sequencing (NGS) assay and fluorescent in situ hybridization (FISH) and single-nucleotide polymorphism (SNP) microarray, respectively.

Aberration	FISH/SNP microarray and NGS assay	FISH/SNP microarray Unique	NGS assay Unique	Sensitivity, % (95% CI*)	Specificity, % (95% CI*)
Overall	483	6	15	0.99 (0.97–1)	0.99 (0.99–1)
Del(1p)	28	1	0	0.97 (0.82–1)	1 (0.97–1)
Gain(1q)	55	1	0	0.98 (0.9–1)	1 (0.96–1)
Del(6q)	35	1	2	0.97 (0.85–1)	0.98 (0.94–1)
Del(8p)	33	0	2	1 (0.89–1)	0.98 (0.94–1)
Del(13q)	77	1	0	0.99 (0.93–1)	1 (0.95–1)
Del(14q)	40	0	5	1 (0.91–1)	0.96 (0.9–0.99)
Del(16q)	42	0	3	1 (0.92–1)	0.97 (0.93–0.99)
Del(17p)	26	0	2	1 (0.87–1)	0.98 (0.95–1)
HRD	73	0	0	1 (0.95–1)	1 (0.96–1)
t(4;14)	13	0	0	1 (0.75–1)	1 (0.97–1)
t(6;14)	1	0	0	1 (0.03–1)	1 (0.98–1)
t(8;14)	8	2	1	0.8 (0.44–0.97)	0.99 (0.96–1)
t(11;14)	48	0	0	1 (0.93–1)	1 (0.97–1)
t(14;16)	2	0	0	1 (0.16–1)	1 (0.98–1)
t(14;20)	2	0	0	1 (0.16–1)	1 (0.98–1)

*FISH* fluorescent in situ hybridization, *SNP* single-nucleotide polymorphism, *NGS* next-generation sequencing, *CI* confidence interval, *HRD* hyperdiploidy

### Comparison of SNP microarray versus the custom capture NGS assay

Focusing on the CNAs of relevance for multiple myeloma: del(1p), gain 1q, del(6q), del(8p), del(13q), del(14q), del(16q), del(17p), and hyperdiploidy (HRD), there were 384 CNAs identified by both assays, 3 uniquely identified by SNP microarray and 15 uniquely identified by the custom capture NGS assay (Figs. 2 and 3). HRD, defined as extra copies of two or more of the odd chromosomes, was detected in 73 patients with 100% concordance between SNP microarray and our custom capture NGS assay. Del(13q) was the most frequent CNA (50%), followed by gain 1q (36%) (Table 2). Del(17p) was found in 26 patients (17%) by both SNP microarray and the custom capture NGS assay while two additional arm-level 17p deletions were detected uniquely by the NGS assay.

The overall sensitivity and specificity were 99% and 99%, respectively, for our custom capture NGS assay using SNP microarray as reference. Copy neutral LOH was detected using both SNP microarrays and the custom capture NGS assay (CNAs and copy neutral LOH are shown in Supplementary Figs. S5–S7). In addition, of the 55 cases where a 1q gain was identified, more than one extra copy of 1q was detected in eight cases (Supplementary Fig. S8). There was no correlation between sample purity and detection of genomic events in this study, similar to what was observed in CoMMpass and previous whole-genome sequencing studies^40,41^. There were no samples that did not harbor any genomic aberrations; the less common aberrations are specified in the Supplementary Material.

### Mutations, small insertions, and deletions by the custom capture NGS assay

At least one non-synonymous mutation was identified in 132 of 154 (85%) samples. In these 132 samples, a total of 362 non-synonymous SNVs and 51 Indels were detected by our custom capture NGS assay (median 3/sample). We found that 19% and 18% of the samples each harbored *KRAS* and *NRAS* mutations, respectively, and 5% of patients harbored a *BRAF* V600E mutation. *TP53*, *FAM46C*, and *DIS3* were detected in 12%, 8%, and 6% of patients, respectively (Fig. 1, Supplementary Figs. S9 and S10). Moreover, we identified a tumor-specific clonal V(D)J rearrangement sequence in 127 of 154 samples (82%).

**Fig. 5:**
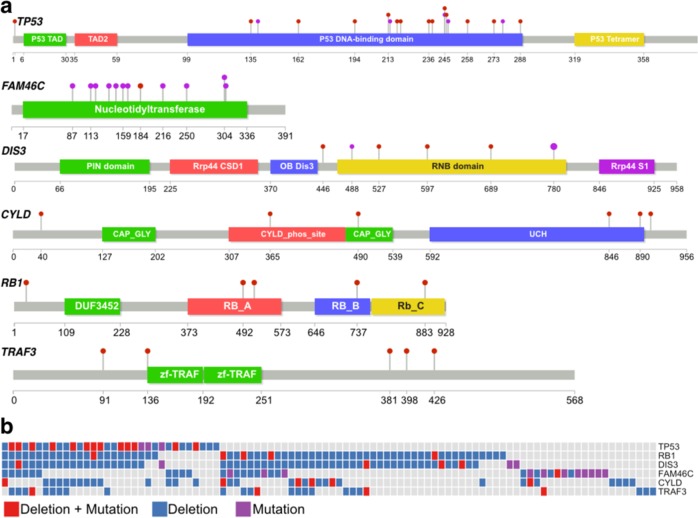
**a** Distribution of somatic mutations in selected tumor suppressor genes. **b** Bi-allelic events detected through sequencing with myTYPE.

With the availability of arm level and focal CNA assessment and mutational data using our custom capture NGS assay, we identified 20 patients with bi-allelic events affecting *TP53, FAM46C, RB1*, and *TRAF3* (Fig. 5). Specifically, among patients with a 17p deletion (*N* = 29), 13 patients (45%) had mutation of the non-deleted allele causing bi-allelic inactivation of *TP53* (Fig. 5).

## Discussion

This is the first large-scale head-to-head comparison of standard of care targeted FISH panel and SNP microarray versus a multiple myeloma designed custom capture NGS assay designed to replace current standard of care prognostic bone marrow assays for multiple myeloma patients with a single test. The study is based on bone marrow specimens from a well-defined cohort of 154 patients with plasma cell disorders. Overall, we found an extremely high concordance (sensitivity > 99% and specificity > 99%) between our custom capture NGS assay and conventional multiple myeloma targeted FISH/SNP microarrays for detection of *IGH* translocations and CNAs. In addition to profiling of *IGH* translocations and CNAs, our custom capture NGS assay captures relevant somatic mutations in multiple myeloma as well as bi-allelic inactivations that are critically relevant for prognostic assessment. Our findings are of high clinical importance as they support the use of one single assay that can replace current standard of care bone marrow assays for multiple myeloma.

For any new assay that is introduced in clinical practice, it should have higher sensitivity and specificity or be more efficient than current options. Multiple myeloma targeted FISH and SNP microarray are labor intensive, require relatively large amounts of bone marrow material, and have limited sensitivity. In this head-to-head comparison, our custom capture NGS assay had a very high sensitivity and specificity in comparison with FISH and SNP microarray in capturing the recurrent genomic aberrations in multiple myeloma. The concordance for the common *IGH* translocations and HRD was 100%. The only translocation that was not fully captured using the custom capture NGS assay was t(8;14) likely due variable *IGH* breakpoints in *MYC* translocations which may partly be outside of the NGS assay primer coverage^42^. Regarding CNAs, our custom capture NGS assay identified a number of additional alterations not detected through SNP microarray analyses.

As for capturing somatic mutations, available NGS panels, such as MSK-IMPACT and FoundationOne CDx, have proven their utility in identifying mutations and CNAs^43,44^. However, none of these NGS panels have been designed to capture *IGH* translocations relevant to multiple myeloma. Because approximately 50% of all patients with multiple myeloma have *IGH* translocations, multiple myeloma targeted FISH and SNP microarrays are still required in parallel with these NGS panels^43,44^. Going forward, it seems logical to conjecture that other groups with interest and expertise in genomics and bioinformatics will develop multiple myeloma designed custom capture NGS assays for clinical use in the future. Also, as new scientific insights become available, there will be need for updated versions of existing multiple myeloma designed custom capture NGS assays.

Analysis of somatic mutations in multiple myeloma is becoming increasingly important for better prognostic assessments and to identify actionable mutations for targeted therapy. In the current analysis, we found that the prevalence of *KRAS* and *NRAS* as well as the overall landscape of somatic mutations were in line with the published literature^5–7^. We conclude that our custom capture NGS assay precisely captures comprehensive genomic abnormalities beyond FISH and SNP microarrays and shows that it is possible to replace current standard of care prognostic bone marrow assays for multiple myeloma patients with a single test.

High-risk multiple myeloma is a relative terminology that is subject to change as modern effective therapies are constantly being improved^10–12^. Per the International Myeloma Working Group consensus criteria, *IGH* translocations t(4;14), t(14;16), and 17p deletions are defined as high-risk multiple myeloma and 1q gains have been included in earlier versions of high-risk definitions^4,45^. More recently, novel potential oncogenes and tumor suppressor genes have been reported in multiple myeloma, e.g. *PTPN11, PRKD2, IDH1/2, HUWE1*, and *UBR5* in addition to the known driver genes in multiple myeloma, i.e. *KRAS, NRAS, BRAF, TP53, FAM46C, DIS3*, and more^8^. Importantly, bi-allelic inactivation of tumor suppressor genes, particularly *TP53*, have been linked to a dismal outcome in multiple myeloma^15^. As illustrated in this study, an advantage of our custom capture NGS assay is the integrated capture of CNAs and mutations co-occurring in the same genes, allowing for the assessment of bi-allelic events involving different types of aberrations. Indeed, in 29 multiple myeloma patients with 17p deletions, our custom capture NGS assay detected *TP53* mutations in 13 patients (45%), resulting in bi-allelic *TP53* inactivation. There is an urgent need for functional studies designed to better understand underlying mechanisms of such cases, which, in turn will allow development of more rational therapies for these patients.

Strengths of this study are the head-to-head comparison of a large patient cohort, and as shown consistently throughout this study, there is high concordance between conventional assays (multiple myeloma targeted FISH panels and SNP microarrays) and our custom capture NGS assay when it comes to detection of *IGH* translocations and CNAs. Limitations include the lack of normal samples for comparison of mutation calling and it imposes challenges for detection of LOH as the inference is based on tumor variant allele frequencies alone. Furthermore, our custom capture NGS assay is designed to capture the IGH locus where >95% of IGH translocations occur which may have impacted the detection of t(8;14) translocations in this study. Also, immunoglobulin light-chain translocations, i.e. *IGK* and *IGL*, may be involved in the pathogenesis in multiple myeloma, however, due to the scarcity of knowledge on their impact, our custom capture NGS assay was not designed to detect *IGK* and *IGL* translocations. Future updated versions of our assay will be revised/expanded in its capture based on biological discoveries.

In conclusion, our multiple myeloma developed custom capture NGS assay captures *IGH* translocations, CNAs, and relevant somatic mutations and thus enables translation of information from recent large sequencing efforts into clinical care. The large head-to-head comparison between targeted FISH panels and SNP microarrays versus our custom capture NGS assay revealed extremely high concordance in regard to detection of relevant *IGH* translocations and CNAs in multiple myeloma. The ability to capture relevant somatic mutations as well as recurrent *IGH* translocations and all CNAs with high sensitivity and specificity in a single assay further supports the clinical strengths of multiple myeloma developed custom capture NGS assays as optimal for patient care in the modern era.

## Supplementary information

Supplementary Material word
