# Correction: Comprehensive detection of recurring genomic abnormalities: a targeted sequencing approach for multiple myeloma

**DOI:** 10.1038/s41408-020-0279-4

**Published:** 2020-01-30

**Authors:** Venkata Yellapantula, Malin Hultcrantz, Even H. Rustad, Ester Wasserman, Dory Londono, Robert Cimera, Amanda Ciardiello, Heather Landau, Theresia Akhlaghi, Sham Mailankody, Minal Patel, Juan Santiago Medina-Martinez, Juan Esteban Arango Ossa, Max Fine Levine, Niccolo Bolli, Francesco Maura, Ahmet Dogan, Elli Papaemmanuil, Yanming Zhang, Ola Landgren

**Affiliations:** 1 Myeloma Service, Department of Medicine, Memorial Sloan Kettering Cancer Center, New York, NY USA; 2 Department of Medicine Solna, Karolinska Institute, Stockholm, Sweden; 3 Cytogenetics Laboratory, Department of Pathology, Memorial Sloan Kettering Cancer Center, New York, NY USA; 4 Bone Marrow Transplant Service, Department of Medicine, Memorial Sloan Kettering Cancer Center, New York, NY USA; 5 Center for Hematological Malignancies, Department of Medicine, Memorial Sloan Kettering Cancer Center, New York, NY USA; 6 University of Milan, Department of Oncology and Onco-Hematology, Milan, Italy; 7 Fondazione IRCCS Istituto Nazionale dei Tumori, Department of Medical Oncology and Hematology, Milan, Italy; 8 Hematopathology Laboratory, Department of Pathology, Memorial Sloan Kettering Cancer Center, New York, NY USA; 9 Epidemiology & Biostatistics, Department of Medicine, Memorial Sloan Kettering Cancer Center, New York, NY USA

**Keywords:** Genetics research, Cancer genomics

**Correction to: Blood Cancer Journal**

10.1038/s41408-019-0264-y published online 11 December 2019

The original version of this Article contained an error in the spelling of the author Ahmet Dogan, which was incorrectly given as Ahmed Dogan. This has now been corrected in both the PDF and HTML versions of the Article.

