# Supplementary material for: Comprehensive detection of recurring genomic abnormalities: a targeted sequencing approach for multiple myeloma
Source: Blood Cancer J. 2019 Dec 11;9(12):101. doi: 10.1038/s41408-019-0264-y (PMC6906304; doi:10.1038/s41408-019-0264-y)
Supplement: Supplementary file 1 — Supplementary Material word [file 41408_2019_264_MOESM1_ESM.docx]

**Comprehensive Detection of Recurring Genomic Abnormalities: A Targeted Sequencing Approach for Multiple Myeloma**

**Supplementary Material**

**Clinical FISH and SNP microarray analysis**

Fluorescent in situ hybridization (FISH) panels for multiple myeloma included probes for t(4;14)/*IGH-FGFR3* fusion, t(11;14)/*IGH-CCND1* fusion, t(14;16)/*IGH-MAF* fusion, t(14;20)/*IGH-MAFB* fusion, *IGH* break-apart, as well as t(6;14) and t(8;14)/*IGH-MYC* fusion probes from Abbott Molecular, Des Plaines, IL and Metasystems, Newton, MA. All FISH testing was performed in the Memorial Sloan Kettering Cancer Center (MSKCC) clinical Cytogenetics Laboratory. Between 100-500 cells, if available, were analyzed and clinical cut-off values for a positive result was 2% for *IGH* translocations. For the comparison, we included *IGH* translocations present in ≥10% of cells in each sample.

Copy number alterations (CNAs) were assessed through single nucleotide polymorphism (SNP) microarray (Affymetrix Cytoscan) with 2.67 million probes including 750,000 common and rare SNP probes. 200ng gDNA was used for the analysis, following the manufacturers protocol. The data analysis was performed combining ChAS 3 software and Nexus copy number software (Biodiscovery, El Segundo, CA) and ASCAT.^1^ Copy Neutral Loss of Heterozygosity (CN-LOH) was reported if the size was at least 10Mb at a terminal region or 20Mb for an interstitial one.

**DNA extraction and sequencing**

DNA was extracted using commercial Qiagen DNA extraction kits. The custom capture next generation sequencing (NGS) assay (myTYPE) uses solution phase hybridization-based exon capture and next generation DNA sequencing to capture all of the recurrent genomic aberrations previously described. Barcoded sequence libraries (New England Biolabs, Kapa Biosystems, Wilmington, MA, USA) were subjected to exon capture by hybridization (Nimblegen SeqCap, Madison, WI, USA). Between 100 to 200 ng of gDNA was used as input for library construction. Libraries were pooled at equimolar concentrations (100 ng per library) and input to a single exon-capture.^2^ To prevent off-target hybridization, a pool of blocker oligonucleotides complementary to the full sequences of all barcoded adaptors was spiked in to a final total concentration of 10 μmol/L. DNA was subsequently sequenced on an Illumina HiSeq 2500 to generate paired-end 101 base pairs for tumor samples and 126 base pairs reads for samples from healthy donors. Sequence data was de-multiplexed using bcl2fastq and sample-wise paired-end fastqs were generated. Short insert paired-end reads were aligned to the GRCh37 reference human genome with 1000 genomes decoy contigs using BWA-mem.^3^

**Genomic coverage metrics**

Overall we obatined a median of 38 million paired end reads per sample across the entire cohort of 154 tumor and 16 nomals samples (Supplementary Figure S1). We obtained an overall median target coverage of 651x per sample (Supplementary Figure S2) resulting from a formidable proportion (median=31.1%) of reads aligning to off-target regions. After marking duplicates, we observed a median of 29.9% duplicates per sample with values ranging from 16.7% to 37.4%.

Next we looked at the coverage across the different regions of target space captured across the bone marrow samples from healthy donors. This analysis was performed using samples from healthy donors alone to prevent for coverage biases caused due to rearrangements and CNAs in the tumor samples. Overall, we obtained a median coverage of 815.6x across the samples from healthy donors. We obtained a median coverage of 750.2x across the coding sequences, 618.1x across finger-printing SNPs, 510.6x for the SNPs in the intronic regions and 914.6x across the *IGH* locus (Supplementary Figure S2a and b). All 16 healthy donors were above 60 years old at the time of collection. The healthy donor samples were analyzed for presence of clonal hematopoiesis; both known clonal hematopoiesis genes and germline predisposition loci were assessed and none were detected.^4, 5^

The percentage of target space with no coverage was overall 0.1% and across the different types of regions with the exception of tiled intronic SNPs (median=18.3%, Q1=18.2%, Q3=18.4) and *IGH* locus (median=3.4%, Q1=3%, Q3=3.7%). The low inter-quartile range of lack of coverage in the intronic SNPs suggests that the same set of SNPs are not being captured by the protocol potentially due to low sequence specificity in these regions.

Across the target space, median of 99% and 98% had atleast 10x and 100x coverage respectively (Supplementary Figure S2c). The same for other captured regions namely exons, fingerprinting SNPs, and *IGH* locus was at least 99% and 97% respectively (Supplementary Figure S2b). The same for Intronic SNPs was 81% and 75% respectively consistent with lack of coverage in 18% of this targeted space.

**Structural rearrangements**

**BRASS:** Given the smaller fragment insert sizes in targeted capture, the 101 bp paired-end reads were trimmed to 50bp from the 3’ end of the read for better discovery of structural rearrangements. Alignment on the trimmed reads was performed as previously described and structural rearrangements were detected by BRASS and Delly.^6, 7^ BRASS first groups discordant read pairs that span the same breakpoint and using Velvet de novo assembler,^8^ then performs local assembly within the vicinity to reconstruct and determines the exact position of the breakpoint to nucleotide precision. Translocations in which either of the break-points was not involved with the *IGH* locus were excluded for downstream analysis.

**Delly:** Additionally, an orthogonal pipeline using Delly^7^ (Version: 0.7.6) was used to identify structural rearrangements. Delly was run on each tumor sample using an unmatched control sample and only somatic calls were retained. Additionally, only those calls having at least 1 spanning read and 1 junction read or at least 4 spanning reads were retained. As previously described for BRASS, translocations should have either of the break-points involved with the *IGH* locus and for deletions, inversions and duplications, neither breakpoint should involve the *IGH* locus.

**Filtering all structural rearrangements**

All calls from BRASS and Delly were further filtered for false positives using average MAPQ, CIGAR Match length, and number of reads supporting the structural rearrangement. MAPQ filter excludes structural rearrangements having average mapping quality of the reads supporting and structural rearrangements with less than a value of 30. CIGAR Match length was the average match length in the CIGAR string of all the reads supporting the structural rearrangement. We used a threshold of <60 to filter calls. Lastly, supporting reads was the number of reads supporting the structural rearrangement. The threshold used for support was at least 30 reads.

Additionally, BRASS and Delly were run on the 16 samples from healthy donors sequenced using our custom capture NGS assay and the post-call filters as described were applied. All structural rearrangements identified in the tumor samples having a break-point detected within +/-50bp of the structural rearrangements in the samples from healthy donors were excluded. The resulting calls retained after the described filters were manually confirmed.

Supplementary Figure S3 and S4 show the t(8:14) and t(11:14) structural variations discussed in the results in the main article.

**Copy number alterations**

For samples with unmatched sample, CNVKit and FACETS were used to identify somatic CNAs in the data.^9, 10^ To negate sample specific biases in CNA analysis, all 16 control samples were combined into a pooled reference. Each tumor sample was then compared with the pooled reference to identify somatic CNAs in each sample. CNVKit corrects for biases in regional coverage and GC content, according to the given reference before calculating the log-ratios between the built pooled reference and tumor. Subsequently, Circular Binary Segmentation (CBS) algorithm was applied to obtain the log2 segment means.

We defined an arm level aberration if the absolute value of log2 segment mean >0.1 and if length of the segment covers at least 10% of the length of the entire arm. To identify focal events in genes we targeted, the segment should overlap the gene boundary, should at most be 10% of the length of the arm and have log2 segmenat mean >0.5. To identify arm level CN-LOH, genotypes and supporting read counts at the 1000 genomes phase 3 SNP loci were called using Samtools.^11^ Heterozygous SNPs were defined as those SNPs having variant allele frequencies (VAFs) between 37.5-62.5 and subsequently heterozygous ratio was calculated for all diploid segments. Heterozygous ratio was defined as the number of heterozygous SNPs to the total number of SNPs. Regions with heterozygous ratio <0.000001 are defined as CN-LOH (Supplementary figure S5 and S5). Supplementry Figure S6 shows an example of a sample with high concordance of CNAs called with SNP microarray and the custom capture NGS assay. Supplementary Figure S7 shows a complex chromosomal abberations including copy number gains, CN-LOH as well as t(8;14) translocation. Supplementary Figure S8 shows patient samples with extra gains of 1q.

**Somatic mutations**

Single Nucleotide Variants (SNVs) were called using CaVEMan, Strelka2, and Mutect2 and small insertions and deletions (Indels) were called using Pindel, Strelka2, and Mutect2.^12-16^ CaVEMan compares sequence data from each tumor sample with an unmatched non-cancerous sample and calculates a mutation probability at each genomic locus. All SNVs and indels that passed by at least one caller were included for downstream analysis and variant annotation was done based on Ensembl v74 using VAGrENT.^17^ To improve specificity, a number of post-processing filters were applied to CaVEMan and Pindel as outlined below.

Post-call filtering of SNVs identified by CaVEMan

- At least a third of the alleles containing the mutant must have base quality >= 25.
- If mutant allele coverage >= 10X, there must be a mutant allele of at least base quality 20 in the middle 3rd of a read. If mutant allele coverage was < 10X, a mutant allele of at least base quality 20 in the first 2/3 of a read was acceptable.
- The mutation position was marked by <3 reads in any sample in the unmatched panel of samples from healthy donors.
- The mutant allele proportion must be >5 times than that in the matched normal sample (or it was zero in the matched normal).
- If the mean base quality was <20 then less than 96% of mutations carrying reads are in one direction.
- Mutations within simple repeats, centromeric repeats, regions of excessive depth (https://genome.ucsc.edu/) and low mapping quality were excluded.

Post-call filtering of Indels identified by Pindel

- For regions with sequencing depth <200X, mutant variant must be present in at least 8% of total reads
- For regions with sequencing depth >=200X, mutant variant must be present in at least 4% of total reads
- The region with the variant should have <= 9 small (<4 nucleotides) repeats
- The variant was not seen in any reads in the matched normal sample or the unmatched panel of samples from healthy donors
- The number of Pindel calls in the tumor sample was greater than 4 and either:
  - The number of mutant reads mapped by BWA in the tumor sample was greater than 0 or
  - The number of mutant reads mapped by BWA in the tumor sample was equal to 0 but there are no repeats in the variant region and there are reads mapped by Pindel in the tumor sample on both the positive and negative strand
- Pindel ‘SUM-MS’ score (sum of the mapping scores of the reads used as anchors) >=150

Subsequently, filtering for known variation and artifacts were applied as described below. All calls retained were manually curated for pathogenicity evidencing existing literature. Genes in the MAPK and NFKB pathways included in the panel are listed in the Supplementary Table.

**Filtering of all SNVs and Indels**

Calls retained after applying the above filters were additionally annotated with variants from MMRF CoMMpass Interim Analysis 9 exomes^18^ (n=889), Bolli et. al. (n=418)^19^ and Lohr et al. (n=203)^20^. Calls were annotated if present at the exact genomic position with the exact mutation of if present in close proximity of a mutation (+-9 bp). For all filtered SNV and Indels identified additional filters were applied and calls were filtered if:

- Present in the *IGH* locus
- Present in a gene not in the panel
- Variant was annotated as a synonymous change
- Minor Allele Frequency (MAF) > 3% in Exac (Version 0.3)
- Filter calls with > 0.5% MAF in Exac (Version 0.3) or 1000 Genomes.
- Present in an unmatched normal sequenced unless present in COSMIC (v81) at the same position or occurring at the very least altering the same amino acid
- Filter missense mutations between 0.0025-0.005, unless these are present in *TP53* or *BRCA1/2* and not present in COSMIC
- Variants are present with reads only on 1 strand
- VAF was less than 3%
- Variants have less than 5 supporting reads
- Target depth of coverage was less than 100x

For both SNVs and indels, variants that may have failed post processing filtering criteria but mapped to recurrent oncogenic mutations in COSMIC^21^ were retained for manual curation.

Supplementary Figure S9 shows all somatic mutations detected with our custom capture NGS assay and Supplementary Figure S10 shows the frequencies of the most common mutations detected with the custom capture NGS assay in relation to the MMRF CoMMpass study. There were no samples that did not harbor any genomic aberrations. The genomic aberrations not included in the figures were less common *IGH* translocations and CNAs, e.g. t(13;14), t(14;15), del2p, del2q, del8p, del8q, del10q, del20p, and 19q gain as well as somatic mutations *KLHF6* (stop gain mutations), *TRAF2* and *PCLO* (missense mutations).

***IGH* V(D)J rearrangement calling**

High sequencing coverage of the entire *IGH* locus provides the opportunity to identify tumor-specific clonal *IGH*-V(D)J rearrangement sequences. To this end, we applied the MiXCR algorithm (v3.0.10) with standard settings to tumor FASTQ files.^22, 23^ Resulting immunoglobulin repertoires were subjected to strict filtering criteria. The most abundant sequence was considered clonal (i.e. tumor-specific) if they made up >25 % of the total IGH V(D)J repertoire, and were supported by more than 10 sequencing reads.

**Supplementary Figure S1 a)** Total number of reads sequenced and polymerase chain reaction (PCR) duplicates per sample and thier relative proportions.

**b)** Mean target coverage across normal and tumor samples sequenced with the our custom capture NGS assay (myTYPE).


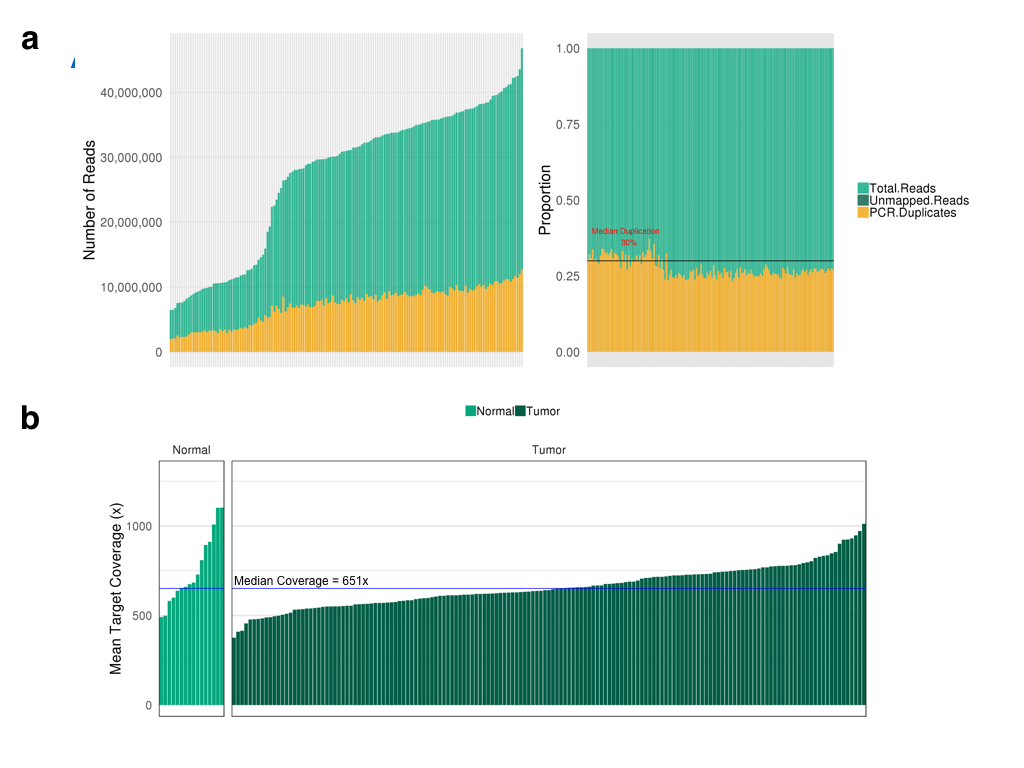


**Supplementary Figure S2 a)** Distribution of coverage across the exons of genes captured **b)** Coverage across the different types of targets captured by the custom capture NGS assay **c)** Percentage of bases with at least 2x, 10x, 30x, 50x and 100x coverage in each target type

**
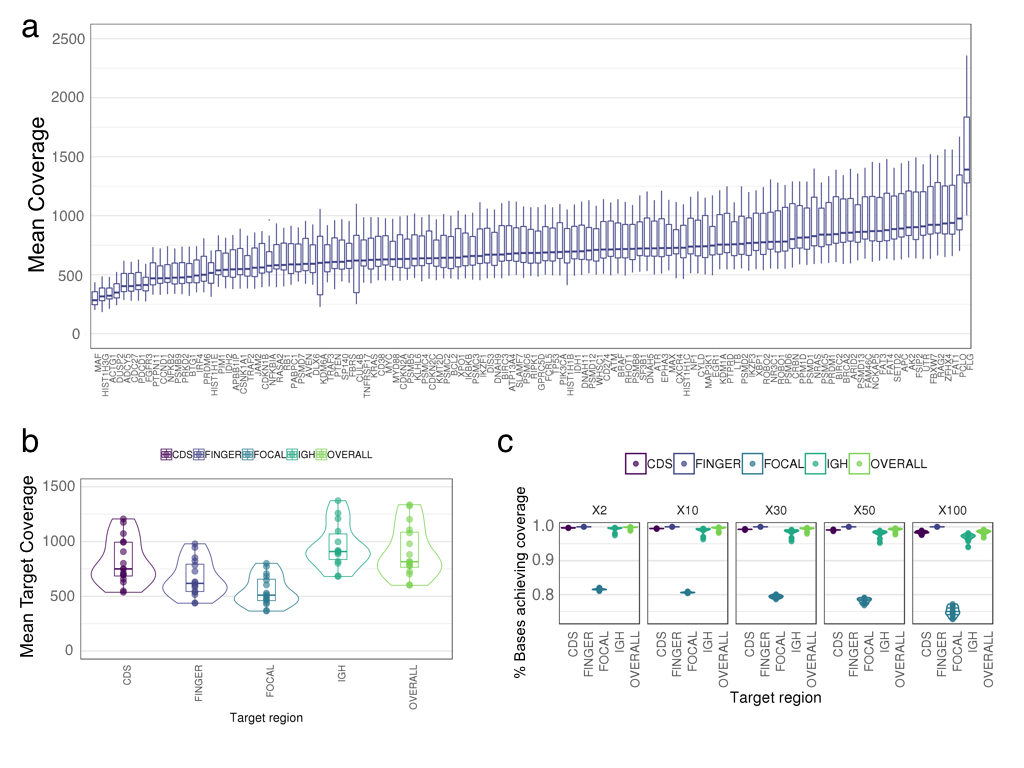
**

**Supplementary Figure S3 a)** Depth of coverage and **b)** aberrantly mapped reads supporting t(11;14) and t(8;14) translocations in on patient sample

**c and d)** IGV snapshots of aberrantly mapped reads supporting **(c)** t(11;14) and **(d)** t(8;14) breakpoints in the same sample


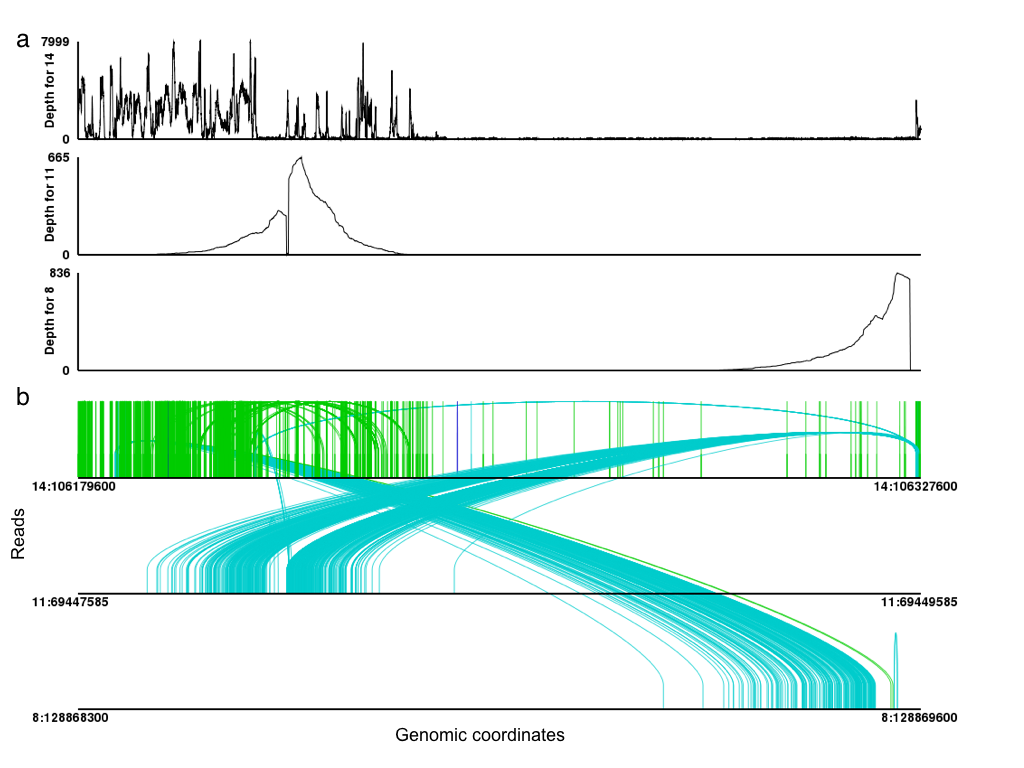


**S3 c and d)**


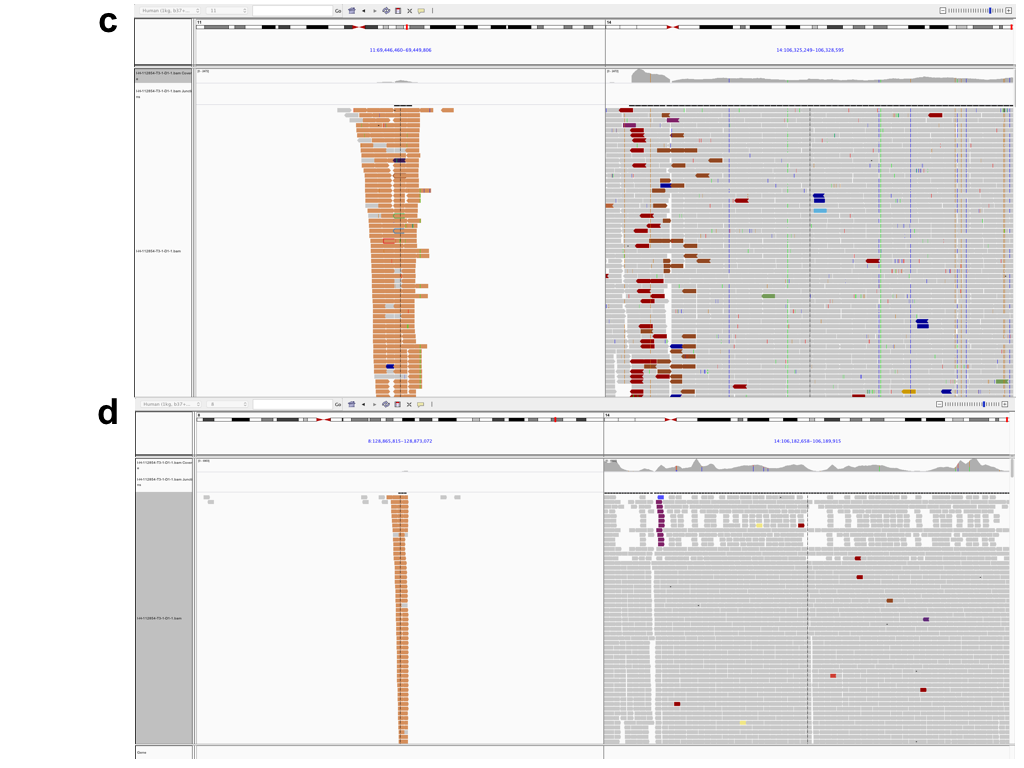


**Supplementary Figure S4.** t(8;14) rearrangements identified in the MMRF CoMMpass IA13 cohort. The location of *IGH* locus captured by the custom capture NGS assay is indicated in red


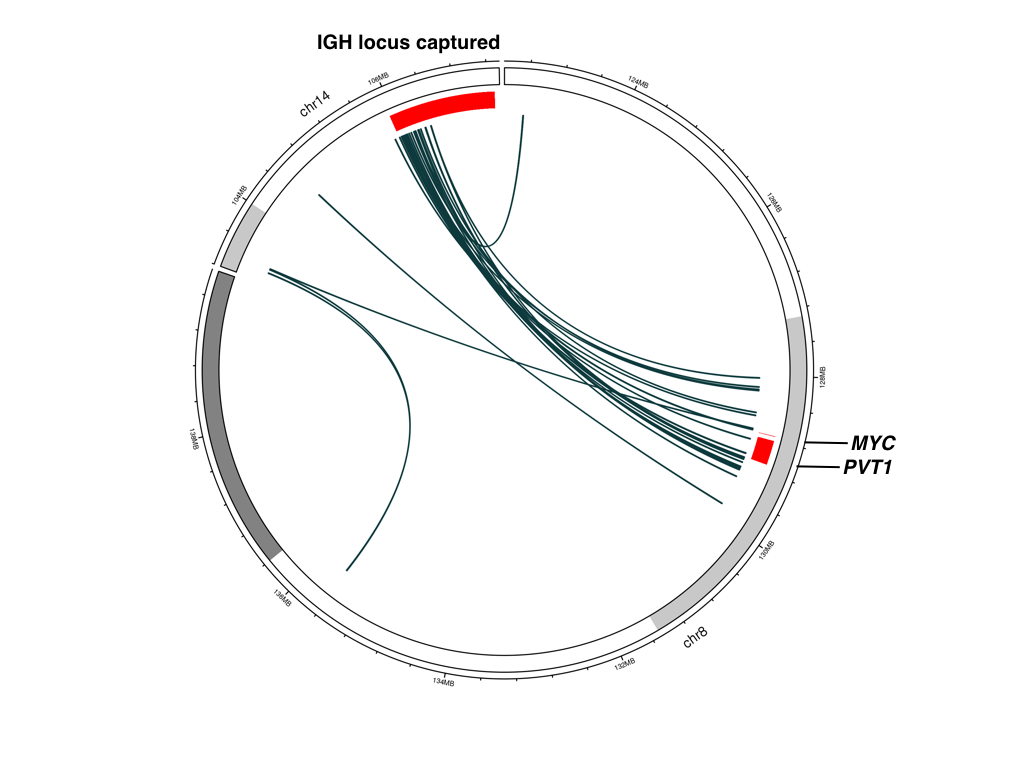


**Supplementary Figure S5.** Detection of copy neutral loss of hetrozygozity (CN-LOH) using the custom capture NGS assay **a)** The log2 copy ratios across the genome for one patient with a diploid state of chromosome 4. **b)** B-allele frequencies (BAF) of the 1000 genome SNPs shows lack of heterozygous SNPs on chromosome 4. **c)** All copy number alterations, including LOH events indicated in green


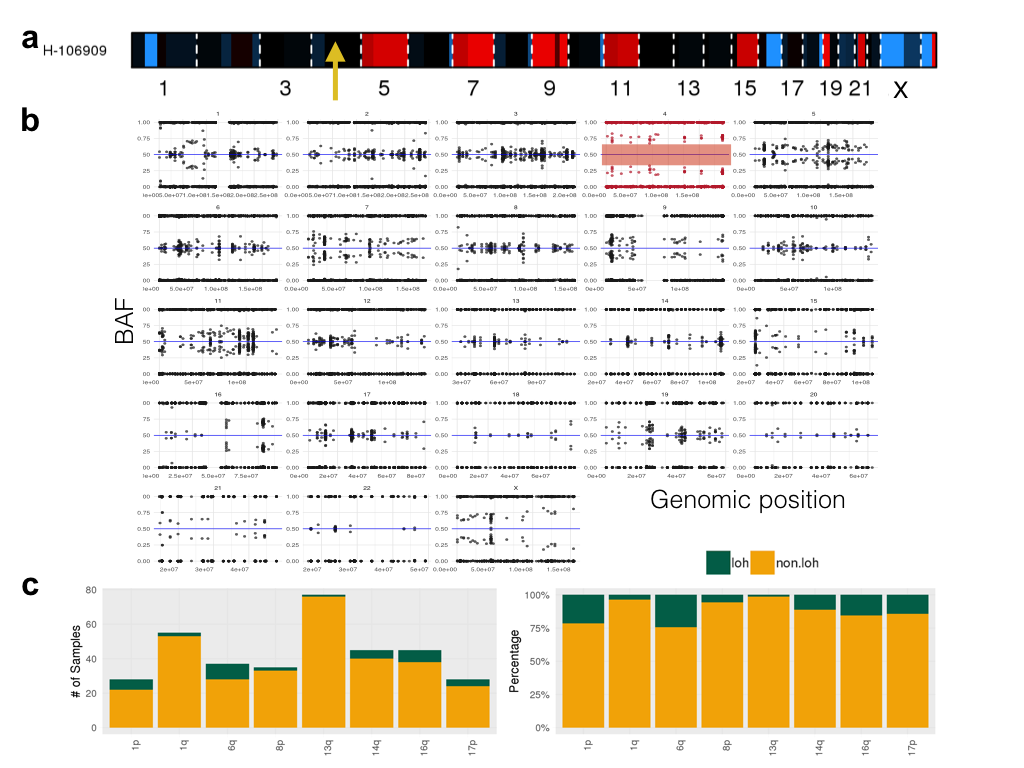


**Supplementary Figure S6**

High concordance of identified copy number alterations exemplified in one patient. **a)** The LogR values in top panel and B-allele frequencies in the bottom panel using ASCAT on SNP microarray **b)** The logR values using CNVKit on sequencing using the custom capture NGS assay.


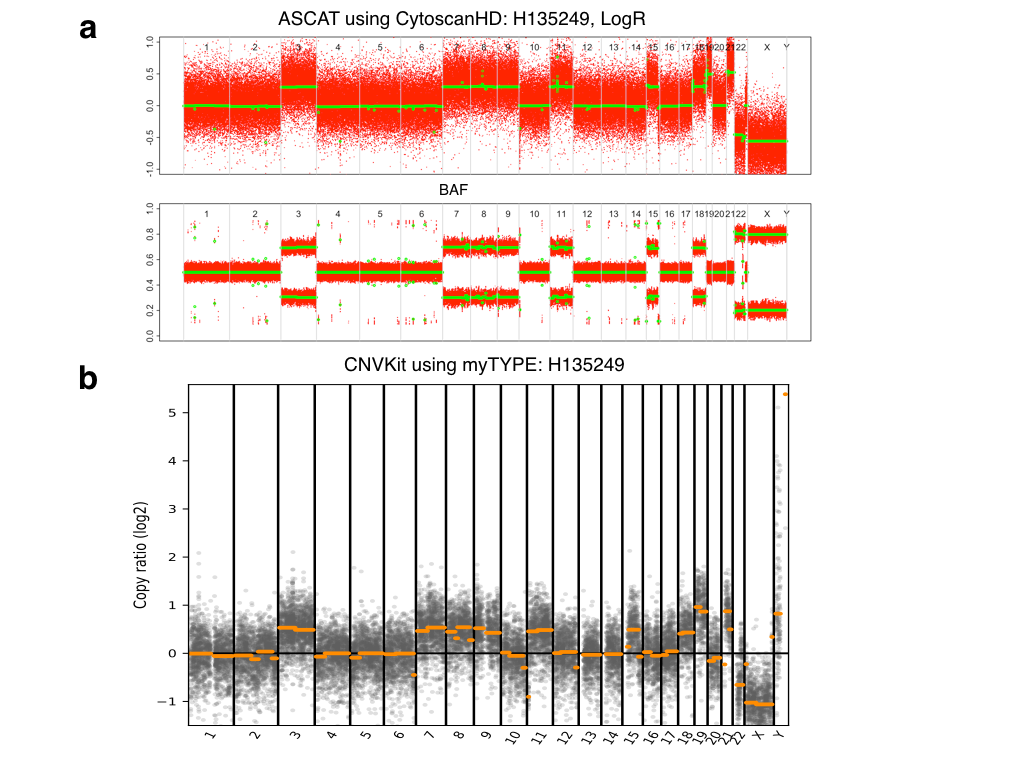


**Supplementary Figure S7a)** One patient sample where FISH shows complex signal patterns with *IGH*/*MYC* fusions in 99% of cells **b)** Characteristic chromosome aberrations in one myeloma patient with gain of 1q, 5p, 6p, 8q (including *MYC*), 7, 9, 11, 15, 18, and deletion of Xp, 4q, 6q, 13, and 17p (TP53)/17q, and CN-LOH of 5q, 14q **c)** terminal gain of 8q with breakpoints at the *MYC* locus d) gain of 14q, CN-LOH, terminal deletion including *IGH*


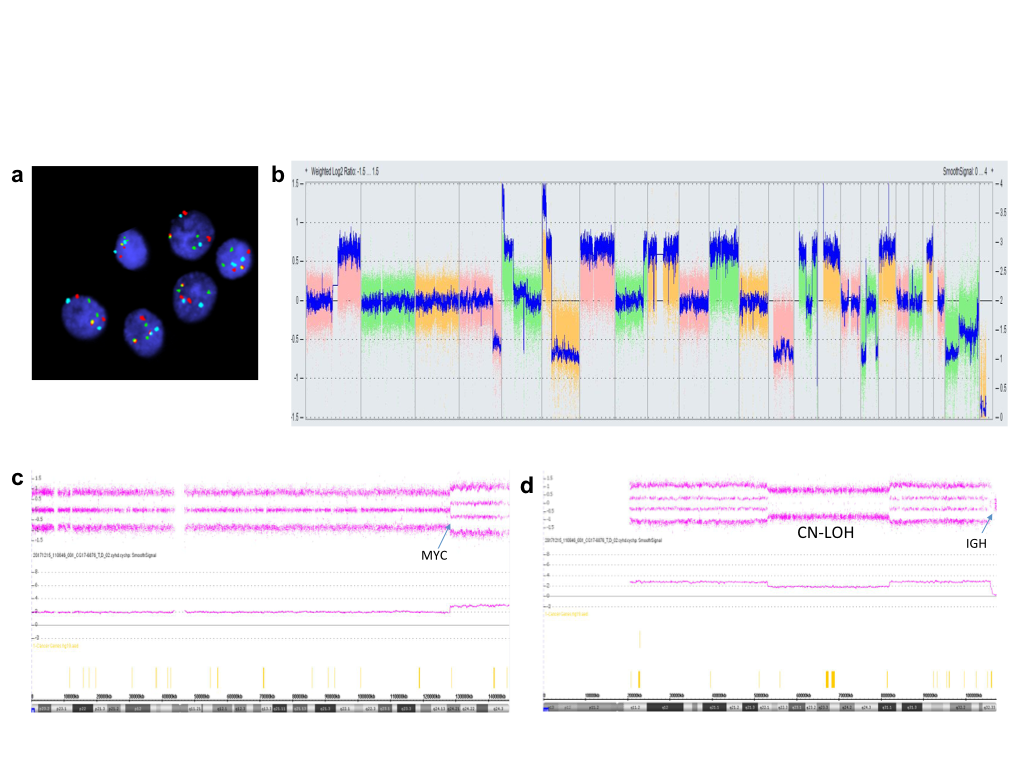


**Supplementary Figure S8** Copy number alterations in samples with **a)** samples with a single gain of 1q and hyperdiploidy and **b)** samples with double gain of 1q. The segmented logR values are shown with amplifications in red and deletions in blue. The segmented logR values in samples with a single 1q gain are shown to have the same intensity as single copy gains in the odd-number chromosomes associated with hyperdiploidy while samples with a double gain of 1q are shown to have a relative higher intensity.


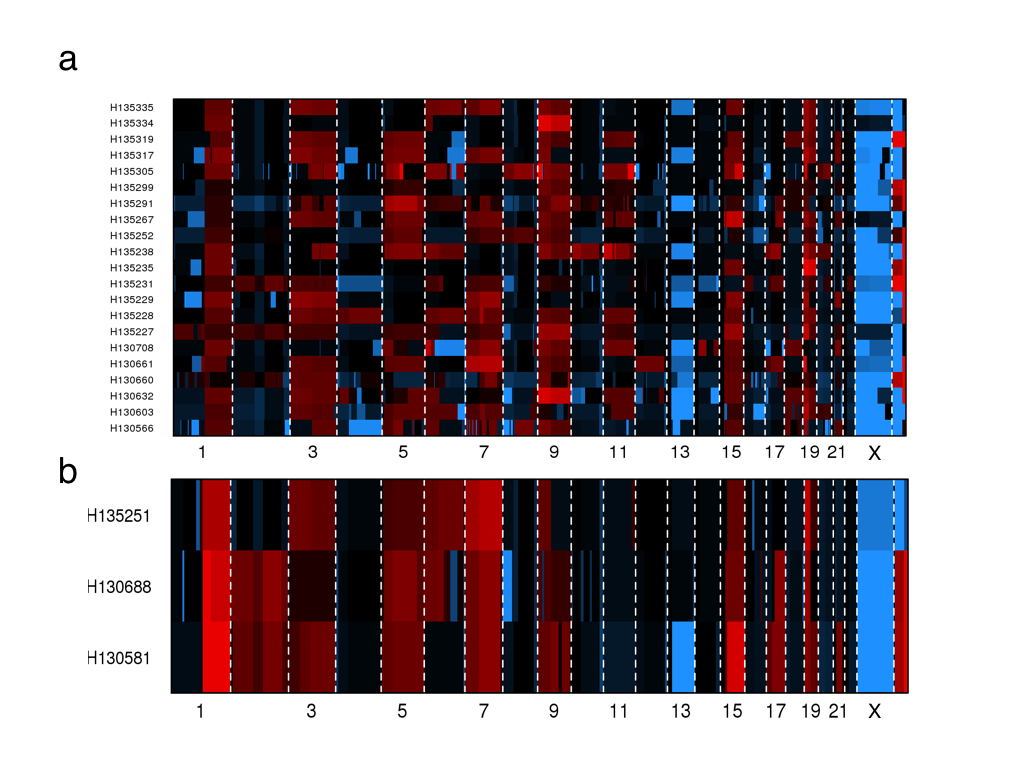


**Supplementary Figure S9** Non-synonymous mutations identified in all 154 samples


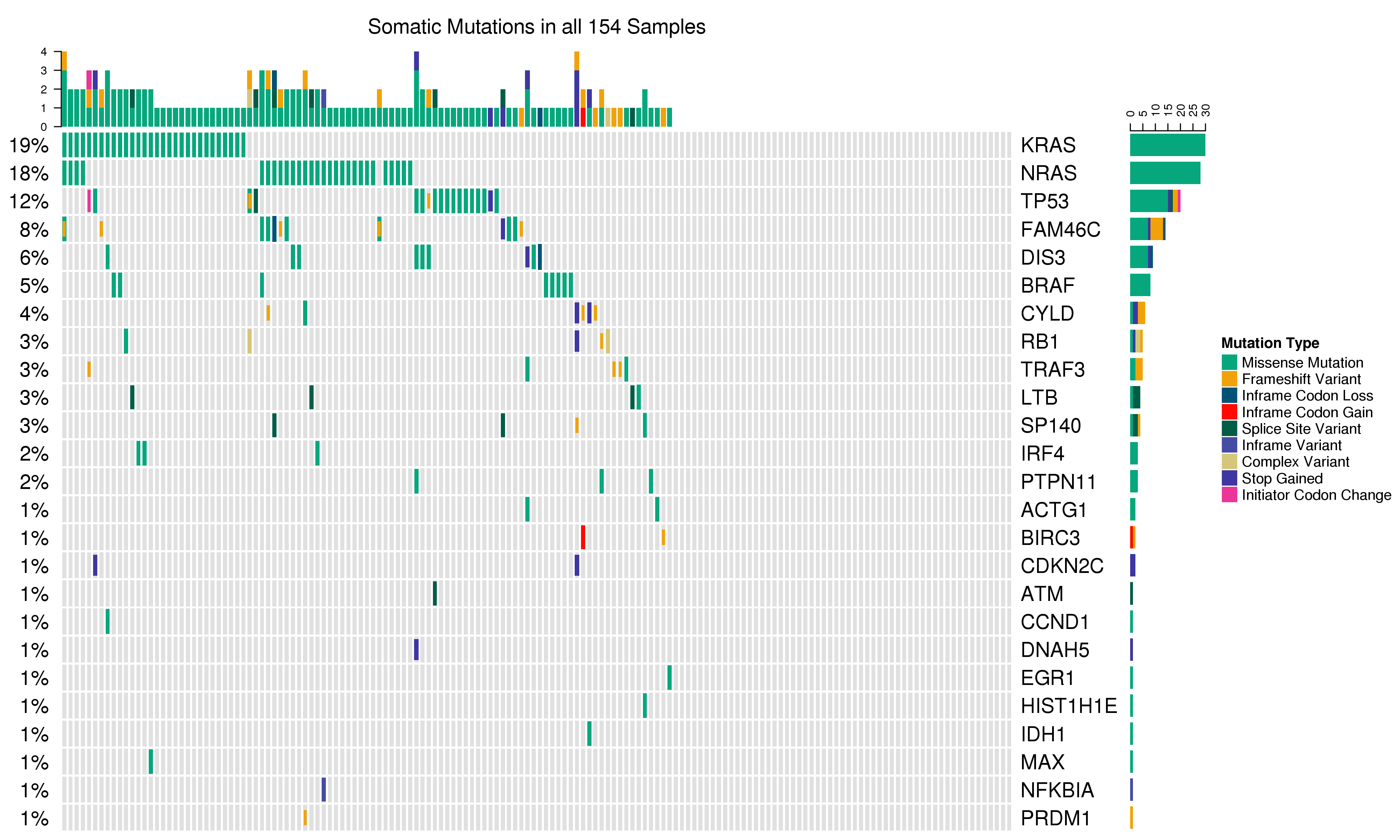


**Supplementary Figure S10** Similar frequency of somatic single nucleotidevariations and small insertions/deletions (SNVs/Indels) in the study cohort as compared to the CoMMpass IA13 cohort


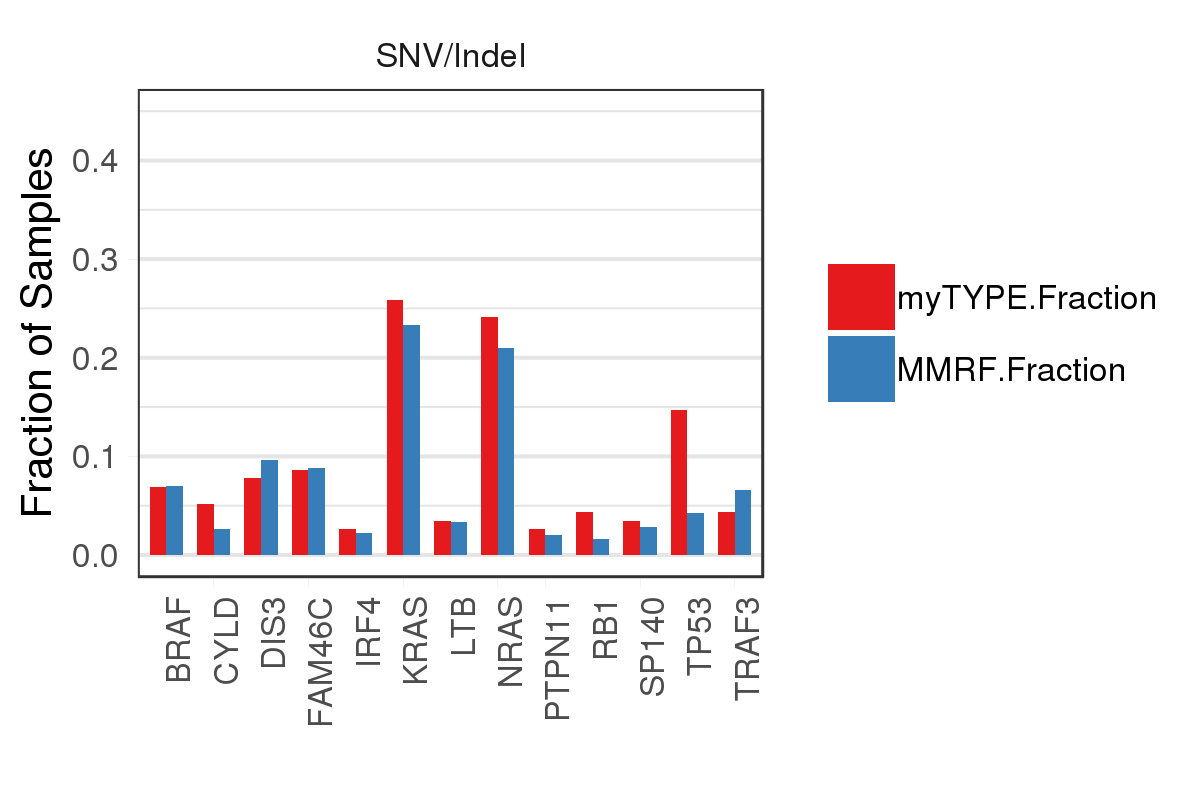


**Supplementary Table.** Genes in the MAPK and NFKB pathways included in the NGS myTYPE assay

| *BIRC2* | *NFKB1A* |
| --- | --- |
| *BIRC3* | *PIM1* |
| *CYLD* | *RIPK1* |
| *IKBKB* | *TRAF2* |
| *IRF4* | *TRAF3* |
| *MAP3K1* | *BRAF* |
| *MYD88* | *NRAS* |
| *NFKB2* | *KRAS* |

**References**

1. Van Loo P *et al.* Allele-specific copy number analysis of tumors. *Proceedings of the National Academy of Sciences* 2010; **107**(39)**:** 16910-16915.

2. Cheng DT *et al.* Memorial Sloan Kettering-Integrated Mutation Profiling of Actionable Cancer Targets (MSK-IMPACT): A Hybridization Capture-Based Next-Generation Sequencing Clinical Assay for Solid Tumor Molecular Oncology. *J Mol Diagn* 2015 May; **17**(3)**:** 251-264.

3. Li H. Aligning sequence reads, clone sequences and assembly contigs with BWA-MEM. *arXiv preprint arXiv:13033997* 2013.

4. Papaemmanuil E *et al.* Genomic Classification and Prognosis in Acute Myeloid Leukemia. *N Engl J Med* 2016 Jun 9; **374**(23)**:** 2209-2221.

5. Papaemmanuil E *et al.* Clinical and biological implications of driver mutations in myelodysplastic syndromes. *Blood* 2013 Nov 21; **122**(22)**:** 3616-3627; quiz 3699.

6. Nik-Zainal S *et al.* Landscape of somatic mutations in 560 breast cancer whole-genome sequences. *Nature* 2016 Jun 2; **534**(7605)**:** 47-54.

7. Rausch T *et al.* DELLY: structural variant discovery by integrated paired-end and split-read analysis. *Bioinformatics* 2012; **28**(18)**:** i333-i339.

8. Zerbino DR, Birney E. Velvet: algorithms for de novo short read assembly using de Bruijn graphs. *Genome research* 2008; **18**(5)**:** 821-829.

9. Shen R, Seshan VE. FACETS: allele-specific copy number and clonal heterogeneity analysis tool for high-throughput DNA sequencing. *Nucleic acids research* 2016 Sep 19; **44**(16)**:** e131.

10. Talevich E, Shain AH, Botton T, Bastian BC. CNVkit: Genome-Wide Copy Number Detection and Visualization from Targeted DNA Sequencing. *PLoS Comput Biol* 2016 Apr; **12**(4)**:** e1004873.

11. Li H *et al.* The sequence alignment/map format and SAMtools. *Bioinformatics* 2009; **25**(16)**:** 2078-2079.

12. Cibulskis K *et al.* Sensitive detection of somatic point mutations in impure and heterogeneous cancer samples. *Nature biotechnology* 2013; **31**(3)**:** 213.

13. Kim S et al. Strelka2: Fast and accurate variant calling for clinical sequencing applications. Nat Methods 2018 Aug; 15(8): 591-594.

14. Raine KM *et al.* cgpPindel: identifying somatically acquired insertion and deletion events from paired end sequencing. *Current protocols in bioinformatics* 2015; **52**(1)**:** 15.17. 11-15.17. 12.

15. Jones D *et al.* cgpCaVEManWrapper: simple execution of CaVEMan in order to detect somatic single nucleotide variants in NGS data. *Current protocols in bioinformatics* 2016; **56**(1)**:** 15.10. 11-15.10. 18.

16. Nik-Zainal S *et al.* The life history of 21 breast cancers. *Cell* 2012 May 25; **149**(5)**:** 994-1007.

17. Menzies A*et al.* VAGrENT: Variation annotation generator. *Current protocols in bioinformatics* 2015; **52**(1)**:** 15.18. 11-15.18. 11.

18. Lonial S *et al.* Interim analysis of the Mmrf Commpass Trial: identification of novel rearrangements potentially associated with disease initiation and progression. Am Soc Hematology; 2014.

19. Bolli N *et al.* Analysis of the genomic landscape of multiple myeloma highlights novel prognostic markers and disease subgroups. *Leukemia* 2018 Dec; **32**(12)**:** 2604-2616.

20. Lohr JG *et al.* Widespread genetic heterogeneity in multiple myeloma: implications for targeted therapy. *Cancer cell* 2014 Jan 13; **25**(1)**:** 91-101.

21. Forbes SA *et al.* The Catalogue of Somatic Mutations in Cancer (COSMIC). *Curr Protoc Hum Genet* 2008 Apr; **Chapter 10**(1)**:** Unit 10 11.

22. Bolotin DA *et al.* MiXCR: software for comprehensive adaptive immunity profiling. *Nature methods* 2015 May; **12**(5)**:** 380-381.

23. Rustad EH *et al.* Stability and uniqueness of clonal immunoglobulin CDR3 sequences for MRD tracking in multiple myeloma. *Am J Hematol* 2019 Oct 1.
